# Case Report: A case of acute anterior wall myocardial infarction caused by stent fracture in the left anterior descending artery

**DOI:** 10.3389/fcvm.2026.1788132

**Published:** 2026-06-08

**Authors:** Haocheng Dong, Xiao Hao, Yige Zheng, Shijie Guan, Shuren Li

**Affiliations:** 1 Department of Internal Medicine, Hebei Medical University, Shijiazhuang, China; 2 Hebei Key Laboratory of Precision Medicine Translational Research on Cardiovascular Diseases, Hebei General Hospital, Shijiazhuang, China; 3 Hebei North University, Zhangjiakou, China

**Keywords:** acute myocardial infarction, coronary stent fracture, drug-eluting stent, optical coherence tomography, PCI

## Abstract

**Background:**

Stent fracture (SF) is a rare but potentially serious complication after percutaneous coronary intervention, which may lead to in-stent restenosis or acute coronary syndrome.

**Case summary:**

A 70-year-old woman with a history of hypertension and diabetes mellitus presented with acute anterior wall myocardial infarction. She had previously undergone multiple stent implantations in the left anterior descending (LAD) artery. Coronary angiography and optical coherence tomography (OCT) demonstrated a complete stent fracture accompanied by neointimal hyperplasia in the mid-LAD segment. A new drug-eluting stent was subsequently implanted at the fracture site, and the patient had an uneventful recovery.

**Discussion:**

This case highlights that long and overlapping stents may predispose to mechanical fatigue and subsequent fracture. Early detection using intravascular imaging modalities such as OCT is crucial for prompt management and the prevention of recurrent ischemic events.

## 1 Background

Percutaneous Coronary Intervention (PCI) is a key therapeutic approach for coronary artery disease, which restores blood flow by dilating stenotic or occluded coronary arteries, thereby effectively relieving myocardial ischemia. The evolution of coronary stents from bare-metal stent (BMS) to drug-eluting stents (DES) has markedly decreased the occurrence of in-stent restenosis, though DES are associated with a higher incidence of stent fracture (SF) compared with BMS (1). SF is defined as a complete or partial discontinuity of stent struts, which may result in in-stent restenosis, stent thrombosis, and acute coronary syndrome (ACS) (2). Although SF is an uncommon complication following PCI, differences in study design and diagnostic techniques may contribute to an underestimation of its actual incidence, which can nonetheless result in serious consequences (3). The present report describes a case of SF in the left anterior descending (LAD) artery leading to acute anterior wall myocardial infarction.

**Figure 1:**
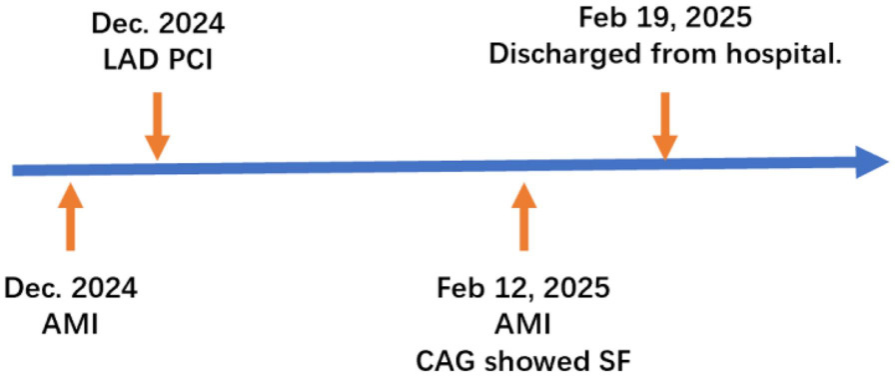
Patient's treatment timeline.

## 2 Case presentation

**Figure 2:**
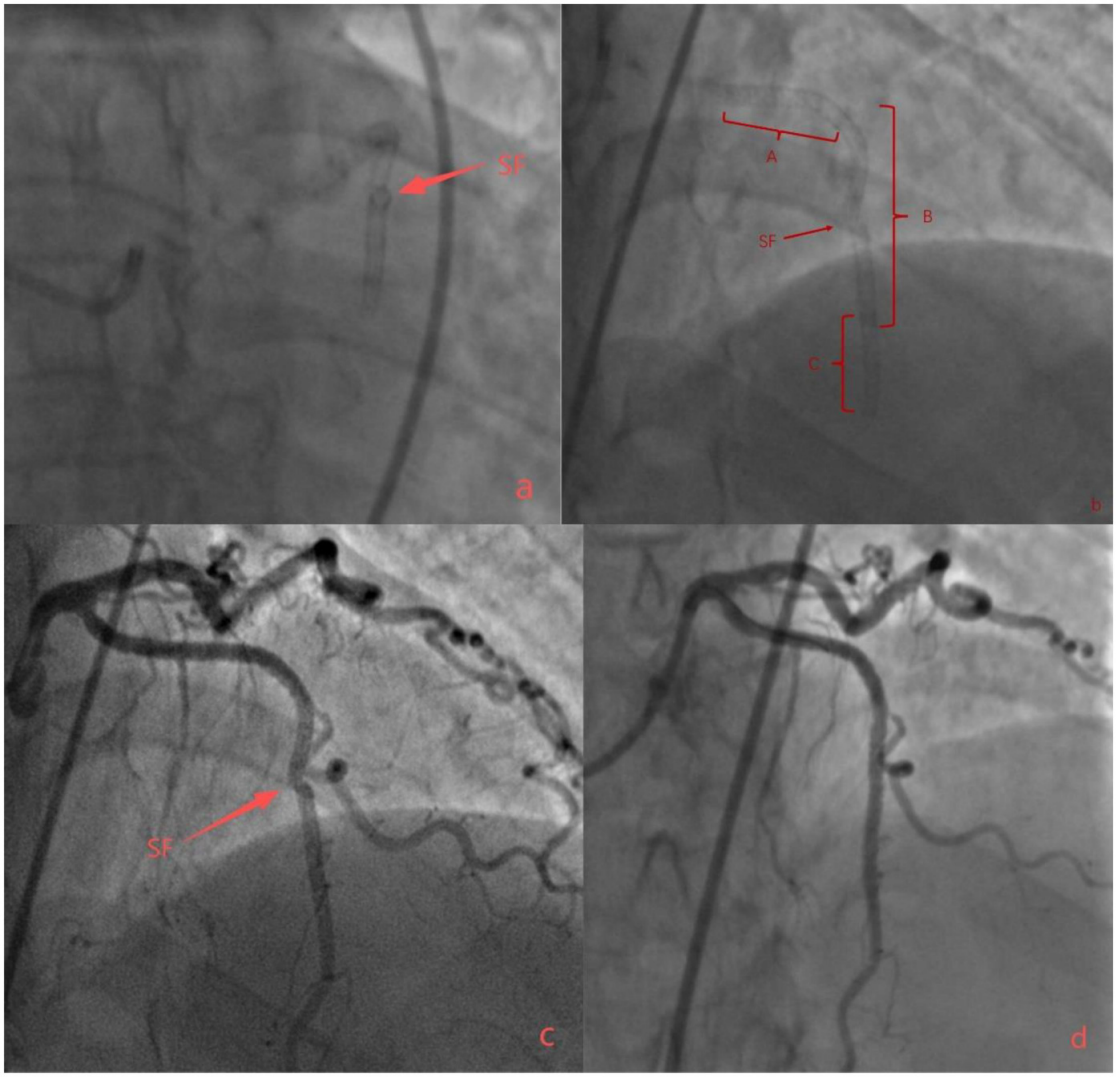
Coronary angiogram performed during this hospitalization. Panels **(A–C)** show fractured stents in the left anterior descending artery; **(A–C)** represent the three stents initially implanted. Panel **(D)** shows follow-up angiography after stent implantation at the site of the stent fracture.

**Figure 3:**
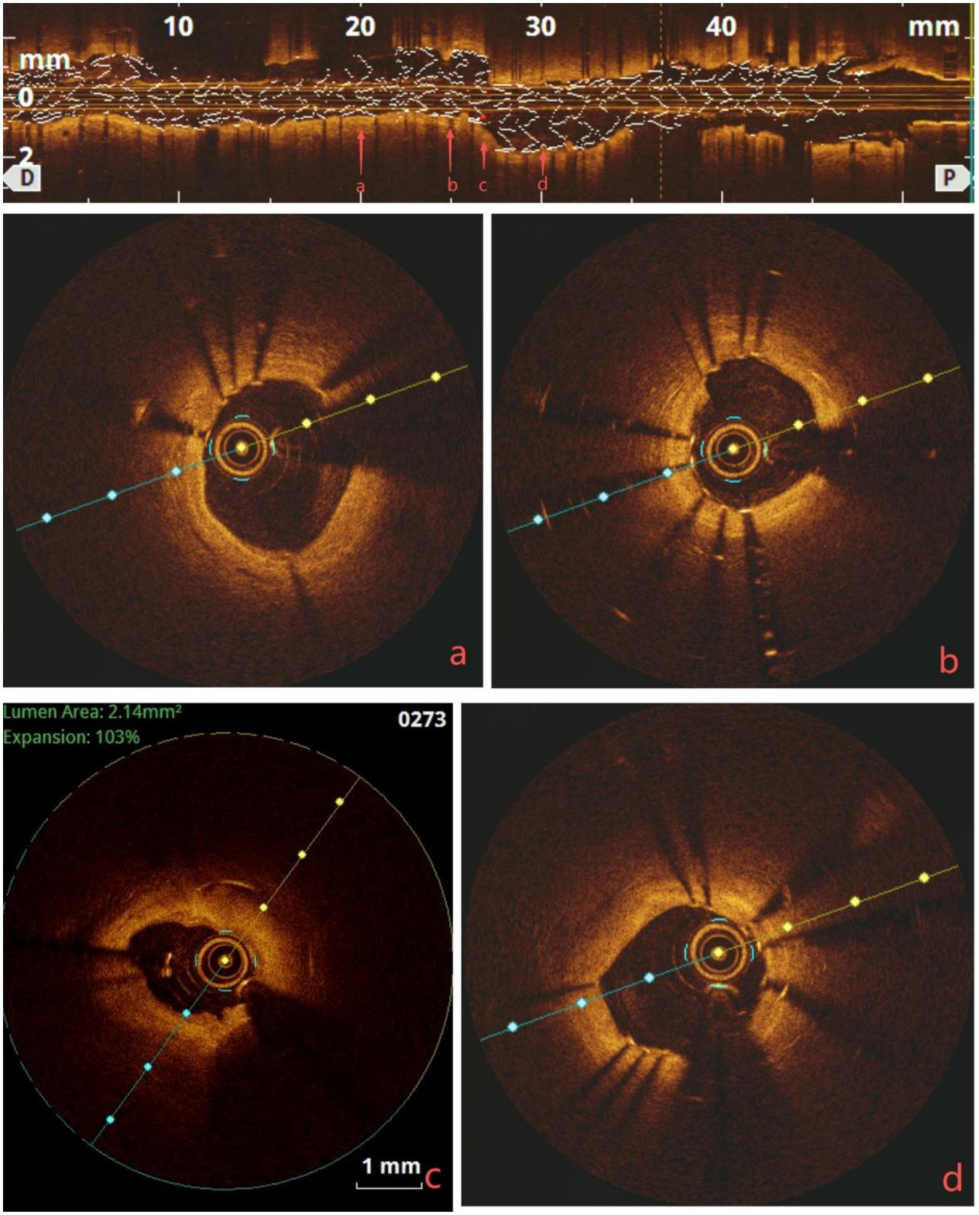
OCT images of the patient. Panel **(c)** illustrates the site of stent fracture, where neointimal proliferation can be observed. Figure **(d)** shows the proximal fracture site, while Figure **(a)** and Figure **(b)** display the distal fracture site.

**Figure 4:**
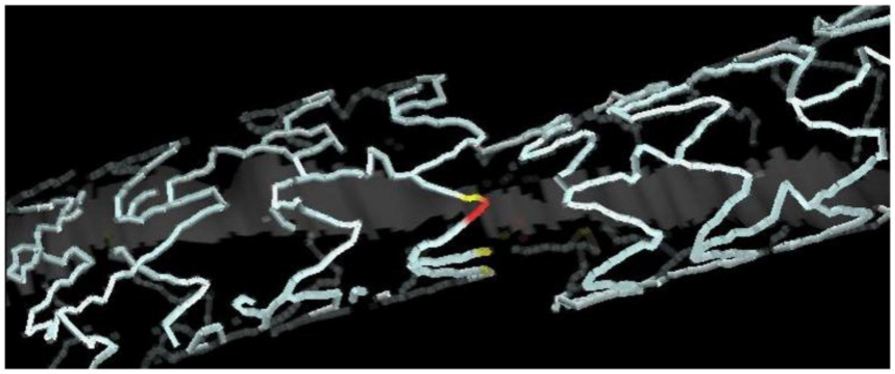
Reconstructed image of the fractured stent obtained by OCT.

The patient was a 70-year-old female, admitted with a chief complaint of paroxysmal shoulder and back pain for 2 months and vomiting for 6 h. In December 2024, she visited a local hospital for shoulder and back pain and was diagnosed with “acute anterior wall myocardial infarction,” but emergency PCI was not performed. One week later, coronary angiography at the local hospital showed occlusion in the mid-segment of the LAD artery with collateral circulation; the mid-segment of the circumflex artery (CX) showed 20%–30% stenosis, and the proximal right coronary artery (RCA) exhibited 30%–50% stenosis with collateral flow to the RCA. PCI of the LAD was performed, successfully opening the vessel. A 2.25 × 15 mm DES was deployed in the distal-mid LAD, followed by a 2.5 × 33 mm DES in the proximal-mid LAD, connected to the previous stent, and finally a 3.0 × 25 mm DES in the proximal LAD, overlapping with the previous one. The procedure was uneventful. Postoperatively, the patient continued treatment with aspirin, ticagrelor, rosuvastatin, evolocumab, metoprolol, spironolactone, and dapagliflozin, without notable discomfort. On February 12, 2025, at 05:50, the patient developed nausea and vomiting without an obvious trigger and presented to our emergency department. She was diagnosed with “acute anterior wall myocardial infarction” and admitted to the cardiology department for emergency PCI. The patient had a history of hypertension and type 2 diabetes mellitus for more than 10 years. Physical examination revealed no abnormal findings in the heart, lungs, or abdomen, and no lower limb edema was observed. Electrocardiography showed sinus tachycardia with ST-segment elevation in leads V2–V5 (HR 110 bpm). High-sensitivity troponin T: 5.802 pg/ml. Echocardiography showed markedly reduced motion in the basal inferior-posterior wall, anterior wall, and apex, with decreased left ventricular systolic function, aneurysm formation at the apex and basal inferior-posterior wall, and grade I–II diastolic dysfunction. On February 12, 2025, coronary angiography (Figure 2) and optical coherence tomography (OCT) were performed, revealing SF with neointimal hyperplasia at the fracture site (Figures 3, 4); a new stent was implanted at the site of fracture. The patient remained clinically stable after the procedure. Following discharge, regular follow-up was conducted in the outpatient clinic, including echocardiography and electrocardiography. Compared with the preoperative status, the patient's cardiac function improved, and no significant ischemic changes were observed on the electrocardiogram. The patient's treatment timeline is shown in Figure 1.

## 3 Discussion

Chowdhury et al. (4) first described a case of SF in 2002. The patient, who presented with chest pain and had previously undergone CABG and PCI, was found on angiography to have a newly occluded saphenous vein graft to the first obtuse marginal branch, with mid-segment fracture of the implanted stent, necessitating repeat surgical revascularization. A meta-analysis reported an incidence of 5.5% for SF in DES (5). Another prospective study involving 6,555 patients with DES implantation detected SF in 12.3% of cases (6). Differences in diagnostic criteria and study design contribute to the heterogeneity of SF incidence. Compared with DES, BMS fractures are less frequently reported. This may be attributed to the lower radiographic visibility of metal density in early coronary angiography, neointimal hyperplasia obscuring fractures, and the limited use of BMS in complex lesions (2). Previous studies have reported that SF can occur as early as 2–7 days after stent implantation and as late as 2 years post-procedure (7). Schochlow et al. classified SF into four types: Type I—single strut fracture; Type II—two or more strut fractures without deformation; Type III—stent deformation; and Type IV—complete transection or misalignment of the stent (8). In this case, the stent in the mid-LAD was found to be fractured with separation and displacement of its two ends, consistent with a Type IV SF.

The risk factors for SF mainly include stent-related and vessel-related factors. The dynamic motion of the coronary arteries exposes stents to continuous biomechanical stress, with metal fatigue being a key contributor to SF. Stainless steel stents are more prone to fatigue and fracture than alloy stents (9). The connectors of a stent may serve as stress concentration points; stents with more connectors are less flexible, resulting in greater stress accumulation at curved segments and an increased likelihood of fracture (9). Long stents and overlapping stents may predispose to SF. Nakazawa et al. analyzed 144 autopsy cases and found a higher prevalence of overlapping stents in SF cases and multivariate logistic regression identified stent length as an independent risk factor for SF (10). One study reported that 65.2% of SF cases involved stents longer than 30 mm, possibly due to increased radial stress in the middle portion of longer stents, resulting in metal fatigue (11). In addition, excessive post-dilation is another risk factor for SF. Data from the MAUDE database indicated that the median post-dilation pressure in SF cases was 18 atmospheres and Chacko et al. found that a stent-to-balloon ratio of 1:1.5 was associated with a higher incidence of SF (11, 12). The discontinued sirolimus-eluting stent shows a higher propensity for SF. In a meta-analysis of eight studies encompassing 108 SF cases, 107 were associated with sirolimus-eluting stents and only one with a paclitaxel-eluting stent,because the closed-cell structure of sirolimus stents results in greater rigidity and susceptibility to fracture (13). In addition, metal components in contemporary coronary stents, such as cobalt, chromium, tungsten, and nickel, may trigger immune reactions and potentially lead to SF (14). Mori et al. reported a case of SF caused by stent hypersensitivity in a 58-year-old man who had undergone three bare-metal stent implantations prior to sudden death. Autopsy revealed focal hypersensitivity reactions at the site of stent fracture in a bare-metal stent located in the right coronary artery (15).

Some vascular characteristics can also elevate the risk of SF. Compared with the LAD and circumflex arteries, the incidence of SF is higher in the RCA, and multiple case reports have documented RCA SF (7, 16). Meta-analyses and retrospective studies also support the finding that SF occurs more frequently in the RCA. The proximal and mid segments of the RCA are typically more tortuous and exhibit greater motion during the cardiac cycle, exposing stents in this region to sustained mechanical stress. Kuramitsu et al. first introduced the concept of “hinge motion,” defined as a change in coronary angulation ≥16° between diastole and systole (17). Their study also showed that the site of SF closely corresponded with the hinge motion point,and the multivariate logistic regression analysis identified hinge motion as an important predictor of SF (17). Schochlow et al. retrospectively analyzed OCT images from three cardiac centers in Germany and found that bifurcation and calcified lesions were more prone to SF,the increased risk is likely due to the structural complexity and altered hemodynamics in bifurcation lesions, as well as inadequate stent expansion in calcified lesions (8). Other vascular factors associated with SF include vessel angulation >45°, tortuous vessels, ostial lesions, and myocardial bridges (2).

Currently, there is no standardized guideline or consensus on the management of SF; prevention and early identification remain crucial. Physicians need to understand the incidence, risk determinants, lesion morphology, and stent characteristics of SF, selecting suitable stents based on lesion-specific factors. Early identification of SF is vital for appropriate management and complication prevention. Given the small size and radiolucency of coronary stents, as well as the angle dependence of 2D fluoroscopy, fractures can easily be overlooked,by comparison, IVUS and OCT provide superior resolution and sensitivity, making them the current gold-standard modalities for diagnosing SF (5). Schochlow et al. classified different degrees of SF based on OCT findings and reported that the detection rate of Type IV SF was ten times higher than that in the control group (8). For asymptomatic SF without signs of in-stent restenosis, conservative management with ongoing dual or triple antiplatelet therapy may be appropriate, though optimal treatment duration remains undefined (18). More severe SF associated with in-stent restenosis necessitates prompt interventional therapy (18). Although overlapping stents are a known risk factor for SF, the priority in severe cases with in-stent restenosis is to restore maximal distal tissue perfusion through appropriate correction of the fracture (19).

## 4 Conclusion

In conclusion, the patient received multiple long, overlapping stents in the LAD artery during the first percutaneous intervention, which likely served as a major risk factor for SF. The diagnosis was confirmed using intravascular imaging, which revealed that the fractured LAD stent had caused in-stent restenosis leading to acute myocardial infarction. A stent was finally deployed at the site of fracture, with the patient demonstrating good postoperative recovery. Therefore, clinicians should minimize risk factors for SF, promptly use intravascular imaging when SF is suspected, and adopt appropriate management strategies accordingly.

## Data Availability

The original contributions presented in the study are included in the article/Supplementary Material, further inquiries can be directed to the corresponding author/s.
